# Meningitis and intracranial abscess due to *Mycoplasma pneumoniae* in a B cell-depleted patient with multiple sclerosis

**DOI:** 10.1007/s10096-024-04935-3

**Published:** 2024-09-13

**Authors:** Dominik Madžar, Florian T. Nickel, Veit Rothhammer, Philipp Goelitz, Walter Geißdörfer, Roger Dumke, Roland Lang

**Affiliations:** 1 Department of Neurology, Friedrich-Alexander-Universität Erlangen-Nürnberg, Schwabachanlage 6, 91054 Erlangen, Germany; 2 Institute of Microbiology - Clinical Microbiology, Immunology and Hygiene, Friedrich-Alexander-Universität Erlangen-Nürnberg, Erlangen, Germany; 3 Institute of Medical Microbiology and Virology, Technische Universität Dresden, Dresden, Germany; 4 Department of Neuroradiology, Friedrich-Alexander-Universität Erlangen-Nürnberg, Erlangen, Germany

**Keywords:** Mycoplasma pneumoniae, Anti-CD20 antibody, Abscess, Multiple sclerosis, Complication, Mastoiditis, Septic cerebral venous sinus thrombosis, Rituximab, Ocrelizumab

## Abstract

*Mycoplasma pneumoniae*, a frequent respiratory pathogen, can cause neurological disease manifestations. We here present a case of *M. pneumoniae* as cause of meningitis and occurrence of an intracranial abscess as a complication of mastoiditis with septic cerebral venous sinus thrombosis in a patient with multiple sclerosis on anti-CD20 therapy.

## Case description

**Fig. 1:**
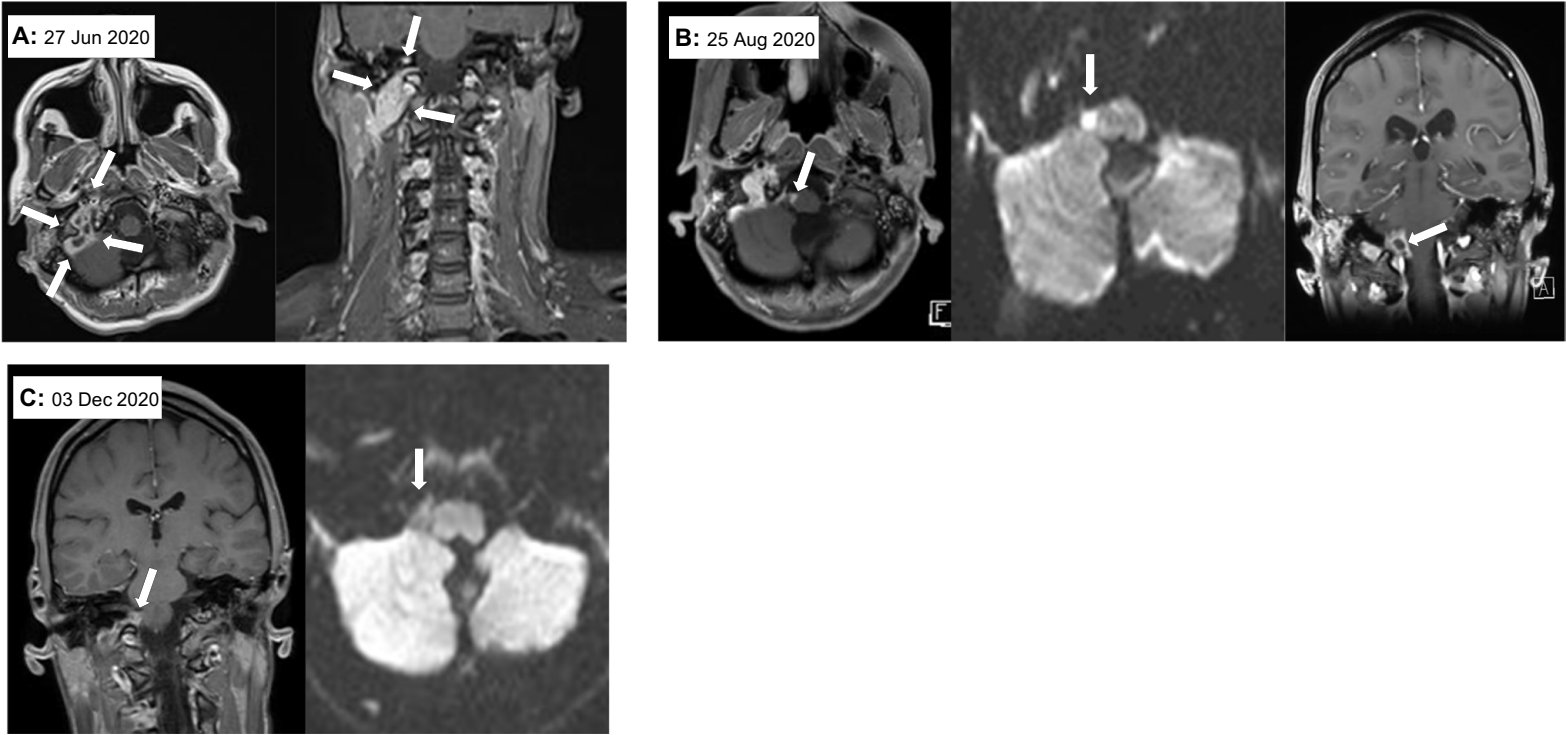
MRI findings: White arrows point **A**: toward the septic thrombosis of the right sigmoid sinus/internal jugular vein at axial T1_tse (left) and coronal T1_tse_dixon with fat saturation (right) post contrast enhancement, **B**: toward an abscess formation adjacent to the medulla oblongata showing circular contrast enhancement at axial T1-3D_MPRAGE (left) and coronal reconstruction of T1_3D_MPRAGE (right) as well as and high central signal on DWI imaging (middle), **C**: toward post-inflammatory scar tissue located in the region of the previous abscess formation that shows no circular contrast enhancement at coronal reconstruction of T1_3D_SPACE (left) and no high central signal on DWI (right) anymore

A 21-year-old female with a history of multiple sclerosis (MS) since 2017 was treated with rituximab (RTX) until 2018 and with ocrelizumab (OCR) thereafter (last dose January 2020). In March 2020, routine MRI showed bilateral mastoiditis, left sternocleidomastoid muscle abscess, and thrombosis of the right sigmoid sinus and right internal jugular vein. Bacteriological cultures from the abscesses and mastoids were sterile, except for skin commensals. Two months later, the patient developed fever and headaches. MRI showed contrast enhancement in the right internal jugular vein and sigmoid sinus (Fig. 1A, [27 Jun 2020]). Cerebrospinal fluid (CSF) analysis findings were consistent with bacterial infection (CSF findings detailed in Table 1). Bacteriological and fungal CSF cultures, as well as 16S rDNA PCR, were negative. Despite multiple antibiotic treatment attempts in the following weeks (including ceftriaxone, piperacillin/tazobactam, meropenem, vancomycin, linezolid, and short courses of clarithromycin, fosfomycin, and doxycycline), the patient’s clinical condition worsened. An FDG-PET yielded pathological FDG uptake only in the right internal jugular vein/sigmoid sinus. A thrombus specimen acquired through catheter angiography from the internal jugular vein was negative by microbiological culture and in 16S rDNA PCR. An MRI for new mild right facial palsy showed a new abscess formation in the right cerebellopontine angle (Fig. 1B, [25 Aug 2020]). The 16S rDNA PCR was positive from a new CSF sample obtained as a consequence of the MRI (7 weeks after the first lumbar puncture), and sequencing of the amplicon identified *Mycoplasma pneumoniae*. This finding was confirmed using *M. pneumoniae*-specific primers for real-time PCR, yielding a threshold cycle (Ct) value of 29.9. Re-analysis of the earlier CSF samples and of the internal jugular vein thrombus, which had been negative in 16S rDNA PCR, demonstrated the presence of *M. pneumoniae* DNA already in the first CSF sample, albeit the late Ct values indicated a much lower bacterial burden. Based on this finding, doxycycline was started. The patient improved clinically and became afebrile. Notably, the patient had already received doxycycline at the beginning of treatment, but only for a few days. Control MRIs revealed gradual abscess regression (Fig. 1C), CSF parameters improved. With a negative PCR result for *M. pneumoniae* DNA in the control CSF analysis and stable MRI findings, doxycycline was discontinued after 151 days. Within 4 weeks the fever recurred. CSF analysis revealed resurgence of even worse infection. MRI confirmed recurrence of leptomeningitis. Doxycycline was restarted in an increased dose (400 mg/d) and levofloxacin was added (1,000 mg/d), resulting in improvement in clinical condition, CSF findings, and MRI. Because of phototoxic skin reaction and worsening of kidney function, the doses of doxycycline and levofloxacin were reduced (200 mg/d and 250 mg/d, respectively). The antibiotic treatment has since been continued as permanent therapy. Neurological examination at the last follow-up visit (May 2023) revealed minor deficits attributable to previous MS lesions and polyneuropathy, most likely after linezolid treatment. Otherwise, the patient was doing well and was leading a normal life.

**Table 1:**
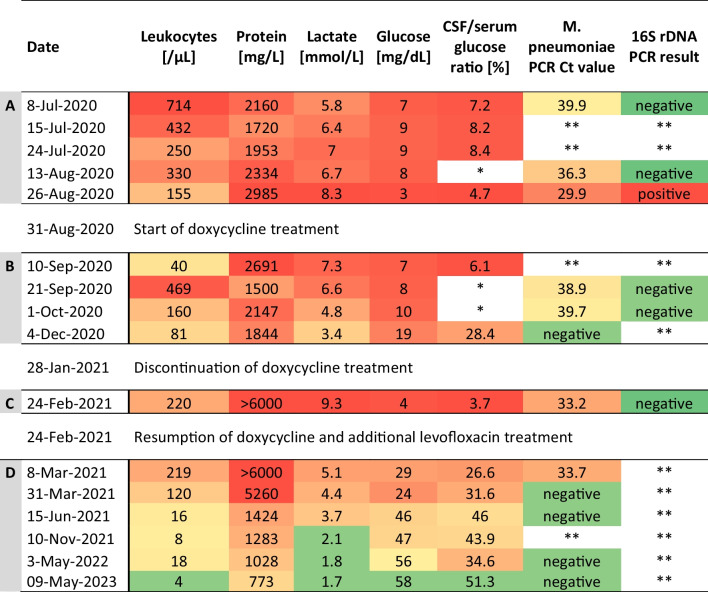
Summary of CSF findings

The table summarizes the CSF findings A) prior to the identification of the causative pathogen, B) after initiation of doxycycline treatment (= phase of marked clinical improvement), C) after discontinuation of doxycycline treatment (= phase of recurrence of fever and clinical deterioration), and D) after resumption of doxycycline and additional levofloxacin treatment. Pathological CSF findings are shown in a color gradient, with increasing shades of red indicating greater deviation from normal values and increasing shades of yellow indicate less deviation from normal values. Normal values and negative PCR results are highlighted in green. *) Missing values because serum glucose was not measured at time of the lumbar puncture. **) Not measured

## Discussion

To the best of our knowledge, we report the first case of meningitis as a complication of septic sinus thrombosis after mastoiditis caused by *M. pneumoniae* associated with B cell depletion by RTX and OCR. We suggest that this case also documents the formation of an intracranial abscess caused by *M. pneumoniae*: Although we have not directly demonstrated *M. pneumoniae* in the abscess formation, the close proximity of the abscess to the infected, thrombotic sigmoid sinus/internal jugular vein and the regression during therapy with doxycycline strongly argues for this interpretation.

In the patient presented, B lymphocytes were undetectable in peripheral blood until 11 months after the last dose of OCR, in line with the time ranges reported in the literature [1]. B cell depletion with anti-CD20 antibodies moderately increases the risk for reactivation of HBV and Herpes zoster, particularly in patients treated for lymphoma and transplant recipients [1]. Overall, the available data suggest that OCR therapy in MS patients does not lead to increased risk of severe infection [2, 3], although the risk for herpesvirus infections was higher [1]. Individual cases of severe infections have been reported after anti-CD20 therapy, including tick-borne encephalitis virus [4] or Candidatus *Neoehrlichia mikurensis* [5]. The identification of *M. pneumoniae* in this case broadens the spectrum of infectious complications after anti-CD20-mediated B cell depletion for autoimmune diseases. Severe systemic infection by *M. hominis* with an abdominal abscess has been reported in a patient with hypogammaglobulinemia due to RTX maintenance therapy of Non-Hodgkin lymphoma [6]. With regard to CNS infections with related pathogens, a case was reported of a brain abscess secondary to mastoiditis caused by *Ureaplasma urealyticum* after combination chemotherapy and RTX for Burkitt’s lymphoma [7].

*M. pneumoniae* is a not infrequent cause of neurological manifestations, including encephalitis (particularly in children), meningitis, ADEM, and Guillain-Barré syndrome [8, 9]. Pathogenesis of CNS disease due to *M. pneumoniae* is not well understood, and may be caused by direct damage of invading bacteria, indirectly by antibody- or T cell-dependent autoimmune phenomena, or by vasculitis and thrombotic vascular occlusion.

The lag period to detection of *M. pneumoniae* in this case was very long, illustrating the diagnostic challenge posed by *M. pneumoniae* as such and in addition by the B cell depletion. As *M. pneumoniae* is difficult to culture, diagnosis is mostly done by serology detecting specific antibodies, which are not induced to significant levels in the patient treated with anti-CD20 antibodies. Pan-bacterial PCR for 16S rDNA were repeatedly negative, despite the confirmed low levels of *M. pneumoniae* DNA in retrospect by specific real-time PCR, demonstrating the limited sensitivity of the broad range 16S rDNA PCR.

Timely diagnosis of *M. pneumoniae* is especially important because of its intrinsic resistance to commonly used broad spectrum antibiotics targeting cell wall synthesis (i.e., of the betalactam and glycopeptide groups). Macrolides have been the drug of choice to treat *M. pneumoniae* for decades because of the low MIC values of wild-type strains, their overall good safety profile, and their immunomodulatory effect that may contribute to clinical improvement in *M. pneumoniae* infections characterized by strong immunopathology. However, macrolides do not penetrate well into the CSF and are therefore not optimal for treatment of meningoencephalitis cases [9]. In addition, there is growing resistance to macrolides among *M. pneumoniae*, especially in Asian countries. In Europe, macrolide-resistance is most prevalent in Italy and France (between 9 and 26%) [10], and around 3% in Germany [11]. Molecular typing of the *M. pneumoniae* DNA isolated from the CSF in this case showed the presence of the A2064G (corresponds to *E. coli* numbering A2058G) substitution in the peptidyl transferase loop of 23S rRNA, which is globally the most frequent mutation associated with resistance to macrolide antibiotics [10, 12]. Since development of macrolide-resistance during therapy has been described [12], it is possible that the intermittent treatment with clarithromycin for one week (last week of July 2020) favored the selection of this macrolide-resistant *M. pneumoniae*. Due to the paucity of specific DNA in the CSF samples obtained prior to therapy with clarithromycin (Ct-values 38.9/39.9), the genotyping for the A2064G mutation could not be performed.

Tetracyclines and fluoroquinolones are treatment choices for macrolide-resistant *M. pneumoniae*. The MIC values for both tetracyclines and fluoroquinolones are higher than for macrolides, but to date no resistance has been reported in *M. pneumoniae* to these groups of antibiotics. In addition, both tetracyclines and fluoroquinolones pass the blood-CSF barrier well and reach therapeutic concentrations required for treatment of meningoencephalitis [13].

Cessation of doxycycline treatment resulted in rapid recurrence and even exacerbation of infection. We hypothesized that in patient the internal jugular vein/cerebral sinus thrombus containing *M. pneumoniae* might serve as a reservoir and a source of bacterial spread if not permanently suppressed by antibiotic treatment. Therefore, we considered permanent treatment warranted, weighing the risks and benefits. Furthermore, the extent of CSF parameter changes we observed are not consistent with previous reports of *M. pneumoniae*-associated meningitis/meningoencephalitis, describing moderate CSF abnormalities [14, 15], whereas massive CSF alterations were rather linked to meningitis/meningoencephalitis caused by *M. hominis* [16].

In summary, this case report illustrates that under B cell depletion unusual infectious agents can lead to severe disease, potentially with unusual presentations and prolonged courses. As this therapeutic principle is increasingly used in autoimmune diseases and more drugs are approved (such as ofatumumab, inebilizumab, ublituximab), the partly atypical infectious side effect spectrum should be kept in mind.

## Data Availability

Due to data regulations, the data used in this study cannot be made available for external use.
